# Building health research systems to achieve better health

**DOI:** 10.1186/1478-4505-4-10

**Published:** 2006-11-06

**Authors:** Stephen R Hanney, Miguel A González Block

**Affiliations:** 1 Health Economics Research Group, Brunel University, Uxbridge, Middlesex UB8 3PH, UK; 2 Center for Health Systems Research, National Institute of Public Health, Av. Universidad 655 Col. Santa Maria Ahuacatitlán, Cuernavaca, 62508 Morelos, Mexico

## Abstract

Health research systems can link knowledge generation with practical concerns to improve health and health equity. Interest in health research, and in how health research systems should best be organised, is moving up the agenda of bodies such as the World Health Organisation. Pioneering health research systems, for example those in Canada and the UK, show that progress is possible. However, radical steps are required to achieve this. Such steps should be based on evidence not anecdotes.

*Health Research Policy and Systems (HARPS) *provides a vehicle for the publication of research, and informed opinion, on a range of topics related to the organisation of health research systems and the enormous benefits that can be achieved. Following the Mexico ministerial summit on health research, WHO has been identifying ways in which it could itself improve the use of research evidence. The results from this activity are soon to be published as a series of articles in *HARPS*.

This editorial provides an account of some of these recent key developments in health research systems but places them in the context of a distinguished tradition of debate about the role of science in society. It also identifies some of the main issues on which 'research *on *health research' has already been conducted and published, in some cases in *HARPS*. Finding and retaining adequate financial and human resources to conduct health research is a major problem, especially in low and middle income countries where the need is often greatest. Research ethics and agenda-setting that responds to the demands of the public are issues of growing concern. Innovative and collaborative ways are being found to organise the conduct and utilisation of research so as to inform policy, and improve health and health equity. This is crucial, not least to achieve the health-related Millennium Development Goals. But much more progress is needed. The editorial ends by listing a wide range of topics related to the above priorities on which we hope to feature further articles in *HARPS *and thus contribute to an informed debate on how best to achieve such progress.

## The need for research *on *research

Health research systems provide a promising opportunity to link knowledge generation with practical concerns to improve health and health equity. Interest in health research, and in how health research systems should best be organised, is moving up the agenda of bodies such as the World Health Organisation (WHO). Pioneering health research systems, for example those in Canada and the UK, show that academic centres and service agencies can be related in ways that encourage the utilisation of research [1,2]. However, radical steps are required to achieve this. These can include establishing virtual national health institutes as a means of promoting networking between existing stakeholders [3]. But implementing such institutional arrangements add further significant challenges to those already confronting many health research systems.

Despite some recent high profile philanthropic donations for health research, many systems face the challenge of constrained resources, both financial and in terms of personnel. Concerns are being raised about not achieving the health-related Millennium Development Goals (MDGs). Other challenges facing health research systems include responding to an increased emphasis on research ethics and developing new ways to commission research so as to allow the greater involvement of patients and the public. There is increased interest in the benefits from health research, but also the need to show value for money and to organise health research systems to achieve maximum payback.

This editorial explains how these and related topics are not just of great significance, but also need themselves to be properly studied. In 1987 Richard Smith from the *BMJ *asserted that there was a 'need to research research' instead of relying on anecdotes [4]. We agree. *Health Research Policy and Systems (HARPS) *aims to provide an outlet for the publication of such research *on *research, and a platform for informed opinions on controversial debates. In addition to identifying topics where it would be useful to initiate new streams of primary research, we also aim to highlight the desirability of bringing existing streams of relevant research to bear on health research systems. In doing so, we draw on a distinguished tradition. Philosophers and researchers in science studies and other disciplines have long addressed issues relevant to current debates about health research policy and systems, including the role of science in society and the desirability and nature of different forms of science [5-8].

Therefore, in what follows we consider the role of *HARPS *in relation to a series of issues. First, we describe the context by: reporting the growing interest at WHO in promoting health research systems; examining some of the current debates within health research systems such as that in the UK; and analysing how such developments fit into wider discussions about the nature of research. Second, we highlight some areas where, we believe, research *on *health research is desirable and show how this can link with existing broader streams of work. Finally, we end the editorial by listing a range of topics which we hope to feature in future *HARPS *articles.

## The context: growing interest in health research systems

In 2001 an international workshop on National Health Research Systems was organised in Thailand with the support of the WHO, the Council on Health Research for Development, the Global Forum for Health Research and the Rockefeller Foundation. Pioneering work was conducted to define health research systems and the strategies needed to strengthen them, and to evaluate their performance [9]. This work was continued by WHO's Research Policy and Cooperation (RPC) department led by Tikki Pang, Founding Editor of *HARPS*. This resulted in a conceptual framework for health research systems [10]. This, in turn, informed the WHO Report, *Knowledge for Better Health*, which focused on bridging the "know do" gap, i.e. the gulf between scientific potential and health realisation. This was seen as central to achieving the health-related MDG's by 2015 [11]. The report was launched prior to the first ever ministerial summit on health research, held in Mexico in November 2004, and the resolutions passed there were endorsed by the 58th World Health Assembly in May 2005 [12].

Related to the resolutions, WHO asked its Advisory Committee on Health Research (ACHR) for advice on ways in which WHO itself could improve the use of research evidence in the development of recommendations, guidelines and policies. This advice was collated by a sub-committee of ACHR chaired by Andy Oxman and is now about to be published as series of articles in *HARPS*, with an introductory editorial by Judith Whitworth, chair of the ACHR.

This exercise illustrates the role *HARPS *can and, we argue, should play. As highlighted in the editorial by Whitworth [13], the work of WHO and its ACHR should herald a major advance in the *use *of research. The published advice will not just take the form of yet another report, it will be published in the open access peer-reviewed literature and will, therefore, be widely available and have been subject to rigorous peer-review. This, in turn, should help to build a culture of research *on *research.

In England, the NHS R&D Strategy developed in 1991 was widely seen as perhaps the first attempt in any country to develop a national R&D infrastructure for the health care system [14,15]. The growing importance attached to health research, and the desire to ensure that benefits are maximised through appropriate organisation of health research systems, are demonstrated by recent events in the UK health research system [16]. No sooner in early 2006 had a review of the National Health Service (NHS) R&D Strategy been completed, the intention to establish a virtual National Institute for Health Research confirmed [3], and implementation started, than another review was initiated – this time of the whole UK health R&D system, with a view to a possible merger between the Medical Research Council and the NHS R&D Programme [17]. The need to 'research research' was highlighted when that second review explicitly stated that, wherever possible, responses 'should be evidence-based'.

One of the tensions identified in the UK (as elsewhere) is the need to find a balance between funding research through independent research councils that have science-led priorities and funding research in response to the priorities of health-care systems. There has been a long debate about how far science should take a utilitarian approach. In the early seventeenth century Francis Bacon advocated harnessing science to meet the needs of society and emphasised the value of synthesising findings [5]. Others have claimed that the best science is produced (and eventually the most good done to society) when scientists determine their own agendas [6,7].

More recently, Gibbons et al. [8] described what they identified as a shift from the traditional discipline-centred mode of knowledge production, that they characterised as *Mode 1*, towards a broader conception, *Mode 2*. In the latter knowledge is generated through a context of application and thus addresses problems identified through continual negotiation between actors from a variety of settings [8]. Another recent conceptualisation, Pasteur's Quadrant, suggests how types of research can be considered according to two dimensions, a quest for understanding and considerations of use. This gives rise to three categories of research depending on the extent to which general understanding arises in the process of solving specific problems, or whether only pure knowledge or pure application is generated. (The fourth category is research that explores particular phenomena without having in view either general explanatory objectives or any applied use) [18].

The context, therefore, is one of growing interest in health research systems, some innovative developments and a well established broader range of analysis, often theoretical.

## Important issues for research *on *health research

Despite the diverse and developing background, there is, as yet, only a limited pool of primary research, as opposed to opinion, on how health research systems can best be organised at an overall level. A few studies have examined both how health-care systems and the research community can work together collaboratively to commission and use research, and how knowledge brokerage roles and receptor functions should be developed [2,19]. And, drawing on Gibbon's characterisation, a recent study reports that health services research involves a mix of *Mode 1 *and *Mode 2 *research [20]. Within WHO, a recent report from the Tropical Diseases Research (TDR) Programme used the categorisation of Pasteur's Quadrant to describe the research conducted by the Programme [21].

At least one body, the Canadian Health Services Research Foundation (CHSRF), is already conducting innovation, evaluation and analysis into ways of organising health research [1]. With the concept of 'linkage and exchange' it has been promoting the importance of collaborative approaches to organising health research systems [22]. It has also promoted the use of knowledge brokers [23]. The 2004 World Health Report and the Mexico summit spurred interest to disseminate and adapt lessons from the CHSRF in the South, and a fruitful collaboration has been started with Mexico and Central America. In many countries it is a challenge to find funding, and/or personnel, for such developments.

Financial and capacity problems impinge more generally on health research systems. Recent generous philanthropic donations from individuals such as Warren Buffett in the USA [24] represent opportunities to address the major health problems of developing countries through appropriate research, but come at a time when concerns are being voiced about not achieving the MDGs and about the stalling of public funding for health research in the USA. However, if increased funding for health research occurs mainly in the North it might draw desperately needed researchers away from the South. Funding increases need to be balanced between North and South if the networks and experience of researchers are to be enhanced globally.

Developing an adequate capacity to conduct research is crucial. And within the South it is particularly important to find ways to attract and retain staff in the public research sector. *HARPS *has published work that shows the need to increase the capacity to undertake research into health policies and systems [25], especially with the increased focus on such topics that arises through the work of the Alliance for Health Policy and Systems Research [26]. Analysis of the capacity of low and middle income countries to undertake equity-orientated research is important [27]. Health research systems in such countries also need to be responsive to growing health problems, such as road traffic accidents, where inter-sector collaboration is likely to be valuable [26,28]. There is also a need to encourage research aimed at assisting the scaling up of health interventions in low and middle income countries [29].

As health research systems develop and respond to demands from the wider societies they encounter difficulties. If health research systems are to develop optimally, it is important that responses to these difficulties are themselves evaluated and analysed. *HARPS *provides a forum for this. For example, there is increased recognition of the need for research to be ethically conducted; it is also important that the roles and impact of ethics committees are evaluated [30]. The development of research agendas and opportunities for an increasing range of stakeholders to influence them are also increasingly being studied and described both in reports [31] and articles [32,33]. There have been many attempts to involve the public in research agenda setting and commissioning, and innovative virtual approaches to commissioning have been evaluated [34]. A recent analysis of how health research findings are communicated explores existing theoretical and disciplinary approaches to the transfer of knowledge; it is important that studies of health research systems build on these [35]. Just as research on health care systems needs to draw on a wide range of disciplines [36], so too research on health research systems will benefit from contributions from various sources.

A recent boost to the production of evidence about health research systems is the start-up funding provided by the Department of Health in England for an observatory of health research systems to be based at RAND Europe. [private communication; Jonathan Grant: jgrant@rand.org]. It is also important to adopt an historical perspective if lessons from previous developments in health research systems are to be absorbed [2,19,22].

There has been considerable analysis, both in reviews [11,37,38] and in primary studies [39], of the many factors that influence the utilisation of health research. Measurement of the benefits of health research – in terms of science *and *of health and wealth – is receiving considerable attention at present. Recent initiatives include those of the European Science Foundation/European Medical Research Councils and of the Canadian Institutes of Health Research. Also a new framework was recently proposed for assessing country-level efforts to link research to action [40].

High profile studies suggest that the benefits from improved utilisation can be enormous, and these studies include ones that attempt to put a value on the health gain that results from research [41,42]. The Funding First study valued the increased longevity in the USA between 1970–1998 at $72 trillion, and claimed that a large part of this could be attributed to health research [41].

There is, plainly, an opportunity for health research systems to provide accountability for funds spent and justification for future funding. The challenge is how to measure benefits in ways that are accepted as being valid and that also meet increasing demands to demonstrate that public funding for research provides value for money, especially when the money could otherwise have been used directly to provide health care. Eye-catching results are sometimes desired. But studies that adopt a more detailed approach, with a multi-dimensional categorisation of benefits and analysis of how specific research might have made an impact [43,44], raise doubts about the applicability of some of the approaches to putting a monetary value on research, especially in systems other than in the USA.

To date most progress in measuring the benefits from research has been made in assessing the impact of research on health policy, either through exploring the impact of specific research programmes [45], or examining policy areas for evidence of policy impact [46,47]. *HARPS *has already published a review of progress and proposals for further action in this field which is often seen as a step towards achieving a health gain from health research [48].

Of particular interest to *HARPS *are studies of the payback *from *health research that can be used to inform policy *on *health research, and indicate how health research systems could best be organised to enhance the payback from health research. Such work has been conducted in the UK, for example on behalf of the Arthritis Research Campaign (**arc**) [43,49]. Commenting on such payback work in the UK, Diane Garnham, then Chief Executive of the Association of Medical Research Charities, was quoted in **arc's **Annual Review as stating 'All medical charities want to improve human health – the difficulty is knowing how to fund research in the right way. I believe it will be patients who will benefit from the Arthritis Research Campaign's leadership in trying to understand this process better' [50].

When WHO/RPC started their deliberations in 2001 on how best to analyse health research systems, one recommendation was that a comprehensive picture of benefits should be developed drawing on examples from many countries. It was proposed that 'an inventory of examples of translation and utilisation' should be collated and organized around a payback framework consisting of impacts of health research on policy, practice and the public and how this led to improvements in health. Such an approach is currently being advocated by WHO. The ACHR argues that: 'Perhaps the most effective way for WHO to gain support among member states, funders, potential partners, and the public for its mandate in health research would be to lead the generation of a substantial body of evidence from evaluation studies showing how using research in policy, programmes, and practices has led to improved health outcomes.' [12].

One of the problems in conducting reviews in this field is the comparative lack of published studies; here the role of *HARPS *in publishing research *on *research should help by providing a vehicle for peer-reviewed open access publications. In some instances studies will already have been conducted, but not published because it was not thought possible to publish them in the peer-reviewed literature. In other cases the studies will not even have been conducted because there is no culture of, or funding for, research *on *health research.

Indeed, in some areas those who make decisions about health research systems are developing policies without the benefit of research *on *research to inform their deliberations. In other areas the existing streams of research *on *research are not sufficiently brought into mainstream arguments about research policy. This is a problem that we hope *HARPS *will go some way to addressing. First, by providing an outlet for the findings of new research *on *health research. Second, by encouraging those engaged in research *on *research to focus more on health research system issues. Third, by bringing the results of this work to the attention of relevant policymakers.

## Future articles in *HARPS*

Following consultation with the editorial board, the editors of *HARPS *will be particularly pleased to publish papers in the following fields:

• How should health research systems be organised?

• How can health research systems help ensure that what researchers do will lead to improvements in health and health equity, especiallyin developing countries?

• Do potential users of research have the capacity and will to make best use of the findings?

• How can health research systems help the process of scaling up health interventions in low and middle income countries?

• Are there means to assess the impact and utility of research?

• What are the social, economic, political conditions affecting the health research community?

• Do researchers have good links with the decision makers and program managers?

• How well informed is the public of the results of research?

• Is the money used to sponsor research spent effectively, equitably and efficiently?

• How can health research systems respond to growing threats to health in low and middle income countries, for example, road traffic accidents?
